# Selection and Validation of Reference Genes for RT-qPCR in Protonemal Tissue of the Desiccation-Tolerant Moss *Pseudocrossidium replicatum* Under Multiple Abiotic Stress Conditions

**DOI:** 10.3390/plants14121752

**Published:** 2025-06-07

**Authors:** Rosa María Nava-Nolazco, Selma Ríos-Melendez, Santiago Valentín Galván-Gordillo, Angélica C. Martínez-Navarro, Mishael Sánchez-Pérez, Rocio Alejandra Chavez-Santoscoy, Martha Bibbins-Martínez, Ignacio Eduardo Maldonado-Mendoza, Analilia Arroyo-Becerra, Miguel Angel Villalobos-López

**Affiliations:** 1 Laboratorio de Genómica Funcional y Biotecnología de Plantas, Centro de Investigación en Biotecnología Aplicada, Instituto Politécnico Nacional, Tepetitla de Lardizábal 90700, Tlaxcala, Mexico; rnavan1800@alumno.ipn.mx (R.M.N.-N.); sriosm@ipn.mx (S.R.-M.); valentingg1984@gmail.com (S.V.G.-G.); acmartineznavarro8486@outlook.com (A.C.M.-N.); mbibbinsm@ipn.mx (M.B.-M.); alarroyo@ipn.mx (A.A.-B.); 2 División de Materiales Avanzados, Grupo de Ciencia e Ingeniería Computacionales, Centro Nacional de Supercómputo, Instituto Potosino de Investigación Científica y Tecnológica, Camino a la Presa San José 2055. Col. Lomas 4 Sección, San Luis Potosí 78216, San Luis Potosi, Mexico; mishael.sanchez@ipicyt.edu.mx; 3 Escuela de Ingeniería y Ciencias, Tecnologico de Monterrey, Av. Eugenio Garza Sada, 2501 Sur, Monterrey 64700, Nuevo León, Mexico; chavez.santoscoy@tec.mx; 4 Centro Interdisciplinario de Investigación para el Desarrollo Integral Regional, Unidad Sinaloa, Instituto Politécnico Nacional, Guasave 81049, Sinaloa, Mexico; imaldona@ipn.mx

**Keywords:** abiotic stress, desiccation, ABA, salinity, osmotic stress, bryophytes, *Pseudocrossidium replicatum*, transcriptome, reference genes, RT-qPCR

## Abstract

Plant abiotic stresses are the main cause of significant crop losses worldwide. The moss *Pseudocrossidium replicatum* is highly tolerant to different types of abiotic stress, such as desiccation. Our group is interested in identifying and characterising differentially expressed genes in response to abiotic stress in this species. However, a collection of validated reference genes for RT-qPCR analysis is essential to normalise the expression of genes in response to the conditions of interest. Here, we assessed 13 candidate reference genes for *P. replicatum* based on their expression stability across transcriptomes from six abiotic stress-related conditions using the RefFinder, BestKeeper, geNorm, and NormFinder programs. The stability and reliability of the proposed reference genes were evaluated under six experimental conditions: control, dehydration, rehydration, abscisic acid (ABA), NaCl, and sorbitol. Interestingly, most proposed reference genes exhibited high stability (low M values) across all analysed abiotic stress conditions. A pairwise variation analysis indicated that only one pair is necessary to normalise RT-qPCR experiments. Each gene was confirmed to normalise the expression of both upregulated and downregulated genes. This represents the first report of validated reference genes for RT-qPCR gene expression studies under abiotic stress in the protonemal tissue of a fully desiccation-tolerant moss.

## 1. Introduction

Plants play a fundamental role in the functioning and stability of ecosystems. Through photosynthesis, they produce the oxygen required by aerobic organisms and form the basis of most trophic chains. They also play a critical role in climate regulation as carbon sinks, accounting for 82% of the plant biomass [1,2,3]. Aside from their ecological importance, plants serve as the primary source of food, fibre, and fuel for humanity [4]. However, climate change is disrupting these essential functions. Decreased rainfall frequency and rising global temperatures have led to ecological imbalances that severely affect crop productivity [5]. Abiotic stress accounts for 70–82% of crop losses worldwide [6,7], particularly in arid and semi-arid regions. Among the various abiotic stressors intensified by climate change, such as heat, cold, salinity, nutrient deficiencies, and toxic metals, drought is the most damaging to agriculture [8,9]. It has caused an estimated USD 30 billion in global crop losses over the past decade [10,11,12,13]. Additionally, the global population is projected to surpass 9 billion by 2050, requiring a 70–100% increase in food production to satisfy rising demand [14]. Simultaneously, the Food and Agriculture Organisation (FAO) warns that, without urgent conservation measures, up to 90% of arable land could be degraded by mid-century [15]. These alarming trends highlight the urgent need to understand how plants respond and adapt to environmental stress, particularly in developing resilient crop varieties capable of withstanding changing climate conditions.

To survive in challenging environments, plants have evolved a variety of adaptations, including stomatal regulation, water storage, the formation of spines as modified leaves, reorganisation of the cell wall, and desiccation tolerance, among others [16,17]. Bryophytes, the earliest land plants [18], exhibit high tolerance to abiotic stress [19,20]. The moss *Pseudocrossidium replicatum*, which belongs to the Pottiaceae family and is widely distributed in central Mexico, has emerged as a promising model for abiotic stress research. *P. replicatum* gametophores can recover Photosystem II efficiency (Fv/Fm) after rapid and prolonged dehydration [21], thereby fulfilling the criteria for “full desiccation tolerance” (FDT) according to the Austin protocol [22]. This ability to withstand desiccation positions *P. replicatum* alongside other FDT mosses, such as *Syntrichia caninervis* and *Syntrichia ruralis* [22,23], which are also members of the Pottiaceae family.

Additionally, our group has gathered clear evidence that in vitro *P. replicatum* protonema cells can restore PSII activity and promote growth after 10 days of exposure to freezing conditions (−80 °C), osmotic stress (2 M sorbitol), and salinity (1 M NaCl) (unpublished results). Unfortunately, the abiotic stress tolerance of in vitro protonema cells has not been reported for *S. caninervis* or *S. ruralis*, with only field-collected gametophores having been extensively studied. Nevertheless, the field-collected gametophores of *S. caninervis* can withstand freezing temperatures (−80 °C for 5 days), UV radiation, and salinity stress (300 mM NaCl) [24,25,26,27].

The most widely studied model moss is *Physcomitrium patens* (formerly *Physcomitrella patens*). As in the case of *P. replicatum*, the availability of an in vitro culture system has allowed the study of abiotic stress tolerance in protonema cells of *P. patens*. However, this species shows limited desiccation and freezing tolerance. For example, it does not survive equilibration at atmospheric water potentials below approximately −13 MPa, so it cannot be considered desiccation-tolerant, and can only withstand desiccation to −273 MPa (13% relative humidity) when treated with ABA [28]. It is notably sensitive to freezing (−4 °C) but is highly tolerant against salinity (350 mM NaCl) and osmotic stress (500 mM Sorbitol) [29,30,31,32].

Although *P. replicatum* is an intriguing species with great potential, it currently has only its chloroplast and mitochondrial genomes sequenced, lacking a fully sequenced genome [33,34]. For newly emerging model species like *P. replicatum*, precise gene expression analysis is essential. Real-time quantitative PCR (RT-qPCR) remains the gold standard for expression analysis studies [35,36] because of its sensitivity, reproducibility, and quantitative accuracy [37]. However, reliability depends on factors such as RNA quality, primer specificity, and particularly the use of appropriate reference genes [38,39]. An ideal reference gene should exhibit stable expression across all treatments, developmental stages, and tissues [40,41,42]. While housekeeping genes like actins (ACT), glyceraldehyde-3-phosphate dehydrogenase (GAPDH), tubulins, ribosomal units (18S or 28S rRNA), and ubiquitin (UBQ) [36,39] have commonly been used, identifying suitable reference genes in mosses presents challenges due to their unique physiology and evolutionary position [43]. Recent studies highlight the importance of validating reference genes specific to each organism and treatment [35,44], as no gene is universally stable [45,46]. Therefore, simultaneous quantification of multiple reference genes is recommended, employing at least two and preferably three reference genes for analysis [38,47,48,49,50].

Several software tools, such as geNorm [49], NormFinder [38], BestKeeper [51], and RefFinder [52], are widely used to assess expression stability. In model and crop species, these tools have identified reliable reference genes. In *Arabidopsis thaliana,* ACT2, ACT7, GAPDH, and UBQ10 are stable across various tissues and under multiple environmental and stress conditions [53,54]; eEF-1α, UBQ10/5, GAPDH, and ACT11 are stable in *Oryza sativa* under hypoxia during germination [55]; 18S rRNA and TUB are stable in *Zea mays* under water deficit and salinity [56]; and multiple genes, including calcineurin B-like protein O2 and ribosomal proteins, ADP-ribosylation factors, GAPDH, and others, are stable in *Solanum lycopersicum* [57].

While interest in bryophytes has increased due to their evolutionary significance and resilience to stress, only a limited number of studies have reported on the validation of reference genes for expression studies. In *P. patens*, traditional genes such as ACT, TUB, EF1α, and 18S rRNA have been evaluated under hormonal treatments in protonema cells cultured in vitro [58]. In *S. caninervis*, α-TUB2 and CDPK demonstrated the greatest stability under abiotic stress, while α-TUB1 and CDPK were suitable for hydration–desiccation cycles; 18S rRNA showed low stability across all evaluated conditions on field-collected gametophores [59]. In a similar study, Park et al. discovered novel transcriptome-derived reference genes (POB1, AKB, UFD2) in *Sanionia uncinata* that showed better performance than traditional genes during cold and drought stress in field-collected gametophores [45].

These findings highlight the need to tailor normalisation strategies to specific organisms and conditions. Furthermore, the disparity in research depth between angiosperms and bryophytes emphasises the importance of validating reference genes in mosses. Given that there are no universally stable reference genes, it is essential to explore normalisation strategies for each organism, tissue, and treatment. Based on the expression stability of transcriptomes under six abiotic stresses, we validated 13 reference genes (some of which have not been described previously) for RT-qPCR in *P. replicatum* under these same abiotic stress conditions. This constitutes the first report of validated reference genes for RT-qPCR gene expression studies in the protonemal tissue of a fully desiccation-tolerant moss subjected to abiotic stress.

## 2. Results

### 2.1. Selection of Candidate Reference Genes from Transcriptome Data

**Figure 1:**
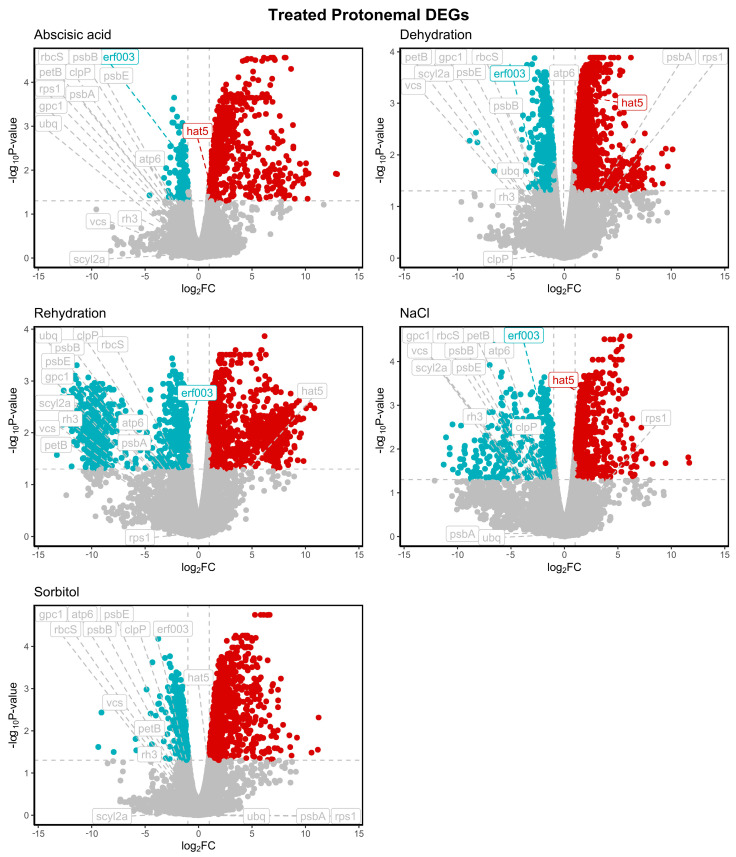
Volcano plots illustrating DEGs under various abiotic stresses and abscisic acid (ABA) treatment in comparison to the control condition. The X-axis represents the log2 fold change between conditions, while the Y-axis displays the −log10 of the adjusted *p* < 0.05. Red dots indicate significantly upregulated genes, whereas blue dots denote significantly downregulated genes.

Using transcriptomes generated from 7-day-old protonemal tissues under control conditions or exposed for 3 h to dehydration (63% HR), rehydration, 10 µM ABA, 200 mM NaCl, or 400 mM sorbitol (unpublished data), we identified the 20 most stable reference gene candidates using BestKeeper and geNorm. It is widely recommended to utilise multiple stability analysis tools simultaneously, as combining various algorithms enhances accuracy thanks to their different evaluation methods [38,49,60]. An in silico curation step was performed to predict the complete ORFs and conserved domains for each gene. Out of the initial set of 20 genes, only 13 met the criteria for completeness and were therefore selected for further analysis. This group included genes encoding Photosystem II protein D1 (*psbA*), Photosystem II protein CP47 reaction centre protein (*psbB*), ATP synthase subunit a (*atp6*), ATP-dependent Clp protease proteolytic subunit (*clpP*), *enhancer of mRNA decapping protein 4* (*vcs*), ribosomal protein S1 (*rps1*), RuBisCO small subunit (*rbcS*), ubiquitin (*ubq*), SCY1-like protein 2A (*scyl2a*), glycerophosphocholine acyltransferase 1 (*gpc1*), DEAD-box ATP-dependent RNA helicase 3 (*rh3*), Photosystem II cytochrome b559 subunit alpha (*psbE*), and cytochrome b6 (*petB*) (Supplementary File S1). Primers for RT-qPCR experiments were designed for 13 candidate genes, and primers for two abiotic stress-responsive genes encoding *a homeobox-leucine zipper protein* HAT5 and *ethylene-responsive transcription factor* ERF03-like proteins were also included (Table 1). Figure 1 shows that clear transcriptional responses were observed across all treatments, with hundreds of differentially expressed genes (DEGs) significantly upregulated (red area; log_2_FC > 1, adjusted *p* < 0.05) or downregulated (blue area; log_2_FC < −1, adjusted *p* < 0.05). As expected for reference genes, all candidates selected in this work remained within the non-significant region of the plot (grey area), indicating no substantial changes in expression (log_2_FC > −1 and < 1, adjusted *p* ≥ 0.05) under any stress condition. This stable expression pattern under various abiotic stimuli supports their suitability as reliable reference genes for gene expression normalisation in *P. replicatum*. Additionally, under dehydration conditions, two genes of interest—*hat5*, a transcription factor upregulated under dehydration, and *erf03*, a transcription factor repressed under the same condition—were identified and selected for validation in RT-qPCR analysis. Their clear differential expression made them ideal targets to assess the robustness of the selected reference genes in subsequent RT-qPCR experiments. The magnitude of expression changes and statistical significance of the selected genes are represented in volcano plots illustrating DEGs for each treatment investigated (Figure 1).

### 2.2. Verification of Primer Specificity

A Primer-BLAST analysis was performed on all primer sets against *P. replicatum* transcriptomes to predict primer specificity, which was further assessed using melting curves, endpoint PCR, and gel electrophoresis analysis. RT-qPCR products for each primer pair amplified only the expected product size, with a single peak observed in the melting curve analysis. The products were visualised on a 2% agarose gel and subsequently verified using RT-qPCR; the dissociation curves for each PCR product revealed a single fluorescence melting peak, indicating that the primers were specific.

### 2.3. Expression Profile of Candidate Reference Genes in Response to ABA and Abiotic Stresses via RT-qPCR

**Figure 2:**
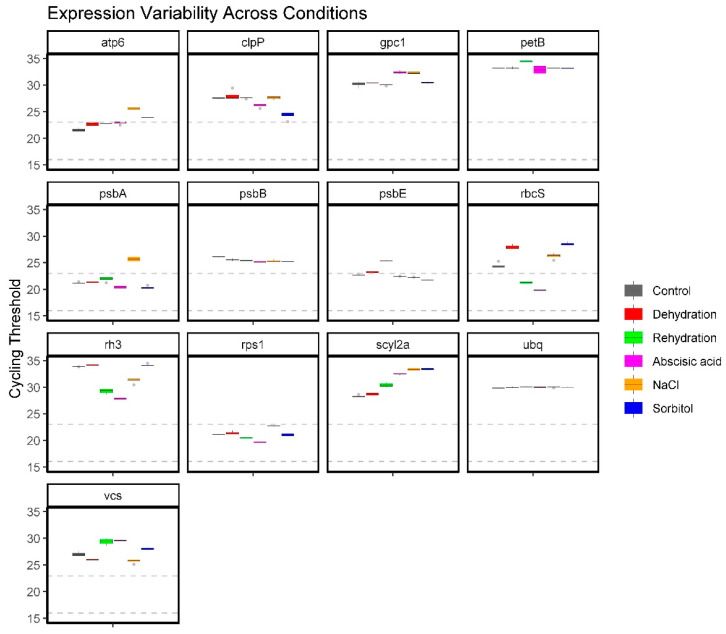
Relative expression levels of 13 candidate reference genes across various abiotic stress conditions and ABA treatment in *P. replicatum* protonemal tissue. Expression was assessed by RT-qPCR and plotted as quantification cycle (Cq) values. Each panel corresponds to a specific gene, with individual box plots representing expression under six conditions: control, dehydration, rehydration, ABA, NaCl, and sorbitol. Dotted lines at Cq 16 and 24 indicate the thresholds proposed for classifying high (Cq < 16), medium (Cq 16–23), and low (Cq > 23) expression levels [61].

cDNA was extracted from the protonemal tissue of *P. replicatum* subjected to various abiotic stress conditions, such as dehydration, rehydration, NaCl, sorbitol, and ABA. RT-qPCR analysis was conducted to assess expression levels using the quantification cycle (Cq) values. These values indicate transcript abundance, with lower Cq values representing higher expression. The Cq profiles displayed a broad spectrum of expression levels across the 13 genes, enabling their classification into low and medium expression categories (Figure 2). Notably, some genes showed consistent expression across conditions, while others exhibited marked variability, underscoring the importance of validating reference gene stability within specific experimental contexts. Among the candidates, *ubq* demonstrated the most consistent and stable expression under all tested conditions, suggesting its suitability as a robust reference gene. Similarly, *psbB*, *petB*, *rps1*, and *atp6* also maintained relatively stable expression profiles across treatments. The *psbA* gene served well as a reference gene except during NaCl stress, when its expression diminished over time. Similarly, *psbE* remained stable in most conditions but showed instability during rehydration. Although *rbcS* is commonly used as a reference gene in other plant models, it displayed significant variability in *P. replicatum* across different treatments. Consequently, *ubq*, *rps1*, *psbA*, *petB*, *atp6*, and *psbB* stood out as the best candidates for reference gene normalisation in RT-qPCR expression studies involving the protonemal tissue of the desiccation-tolerant moss *P. replicatum.*

### 2.4. Evaluation of the Expression Stability of Candidate Reference Genes Using the ΔΔCt Method

**Figure 3:**
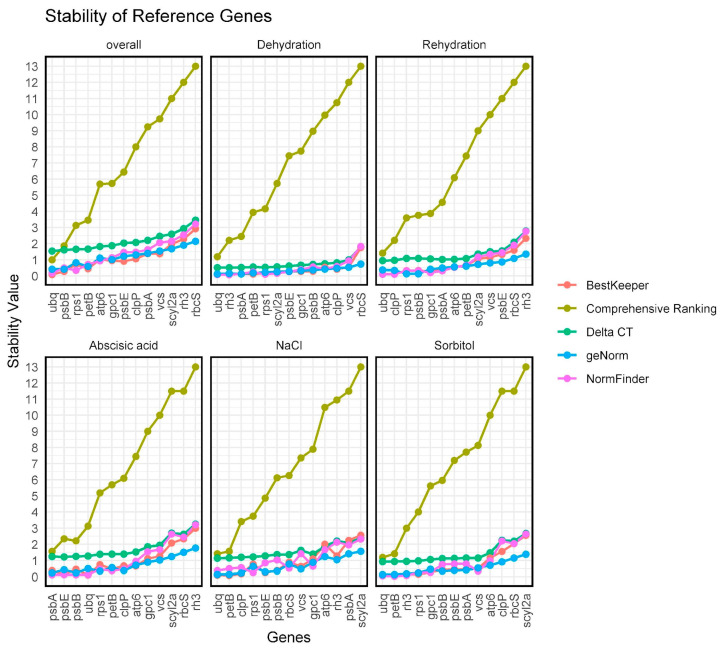
Stability analysis of 13 selected reference genes under abiotic stress and ABA treatments. According to RefFinder, a low average stability value indicates a more stable expression. Genes are ranked from left to right based on their average expression stability, with the most stable genes positioned on the left and the least stable on the right.

To reliably assess the expression stability of the 13 candidate reference genes, we utilised RefFinder, a comprehensive online tool that combines the four most popular algorithms for reference gene evaluation: geNorm, NormFinder, BestKeeper, and the comparative ΔΔCt method. This methodology allowed us to produce individual rankings from each algorithm as well as an integrated overall ranking, based on the geometric mean of the ranks obtained from each method (Figure 3). Stability rankings were independently generated for each treatment condition (dehydration, rehydration, NaCl, sorbitol, and ABA), alongside a unified global ranking across all conditions (Figure 3). For each condition analysed, an independent stability ranking was created, along with a global ranking for all treatments. The findings indicated variability in the stability of the assessed genes based on the treatment, ranging from most stable to least stable. For dehydration treatment, the optimal pair of candidate reference genes was *ubq* + *rh3*, while for rehydration, the best pair was *ubq* + *clpP*. Moreover, the optimal pair for ABA treatment was *psbA* and *psbE*, and for NaCl, it was *ubq* + *petB*. Lastly, for sorbitol, the recommended pair of candidate reference genes was *ubq* + *petB*. It is important to note that some genes displayed variable expression across treatments. For instance, *rh3* ranked among the most stable genes in dehydration and sorbitol, yet it was the least stable in rehydration and ABA. *psbA* demonstrated good stability in ABA but lacked it in NaCl. A comprehensive analysis integrating data from all methods was also included. The global analysis proposed the following ranking from most to least stable: *psbB* and *ubq*, *rps1*, *petB*, *atp6*, *gpc1*, *psbE*, *clpP*, *psbA*, *vcs*, *scyl2a*, *rh3*, and *rbcS*. Based on these findings, it was advisable to use *psbB*, *ubq*, *rps1*, and *petB* as internal controls for normalising gene expression data across all evaluated abiotic stresses.

### 2.5. Evaluation of the Ideal Number of Reference Genes for Normalisation

**Figure 4:**
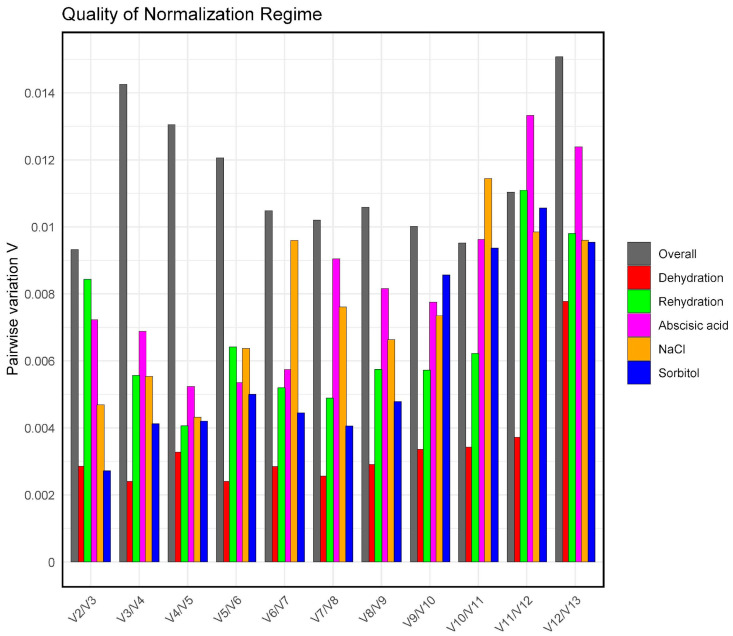
Analysis of pairwise variation (V) utilising the geNorm algorithm aims to identify the optimal number of reference genes for precise normalisation. The Vn/Vn+1 values indicate enhanced expression stability with the addition of another gene.

To determine the optimal number of genes needed for reliable normalisation, we used the geNorm tool. It employs pairwise variation analysis to calculate the Vn/Vn+1 value, which compares the stability of using n genes versus n+1 genes. A cutoff value of 0.15 is suggested [49]: if Vn/Vn+1 < 0.15, no additional genes are necessary; however, if Vn/Vn+1 ≥ 0.15, it is advisable to include more genes for normalisation. The results show that the V2/V3 values for global, dehydration, rehydration, ABA, NaCl, and sorbitol were 0.009, 0.002, 0.008, 0.007, 0.004, and 0.002, respectively (Figure 4). These values are below the threshold of 0.15 recommended by Vandesompele et al. [49]. This suggests that two reference genes were sufficient for robust normalisation under the experimental conditions tested. Consequently, it was advisable to utilise two genes to enhance the reliability of gene expression analyses.

### 2.6. Validation of the Recommended Reference Genes

**Figure 5:**
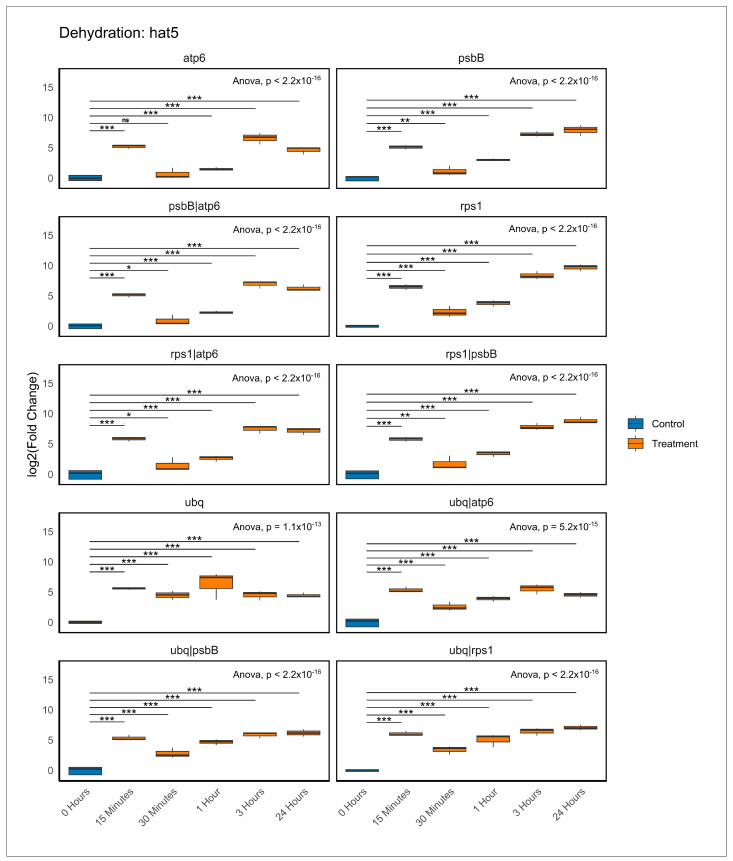
Kinetics of *hat5* gene expression changes under dehydration stress. Box plots represent log_2_ (fold change) values obtained using the ΔΔCt method [62] at five time points: 0 h (control), 15 min, 30 min, 1 h, 3 h, and 24 h of dehydration. Each plot corresponds to a different normalisation strategy using one or two reference genes (*atp6, psbB*, *rps1*, *ubq*). Technical triplicates per sample were included. Statistical significance was assessed using one-way ANOVA followed by Tukey’s HSD test. Asterisks indicate significance levels: *p* < 0.05 (*), < 0.01 (**), < 0.001 (***); “ns” indicates no significance.

To confirm the optimal reference genes identified through our transcriptomic and stability analyses and to deepen our understanding of gene expression during dehydration, we examined the expression profiles of two transcription factors that exhibited contrasting regulatory behaviours. *Hat5* was found to be upregulated in the dehydration transcriptome, while *erf003* was observed to be downregulated (unpublished data). Gene expression was assessed using RT-qPCR at multiple time points (0 h, 15 min, 30 min, 1 h, 3 h, and 24 h) with several normalisation strategies. We utilised individual reference genes (*atp6*, *psbB*, *rps1*, *ubq*, *petB*, *psbA*) and their pairwise combinations to determine which ones most effectively and consistently captured the expected changes in the target gene expressions. In Figure 5, we evaluated the stability of several candidate reference genes (*atp6*, *psbB*, *rps1*, *ubq*, and their combinations) by normalising the expression profile of the transcription factor *hat5*, which was induced by dehydration at different time points (0 h, 15 min, 30 min, 1 h, 3 h, and 24 h). Under control conditions (0 h), *hat5* exhibited low expression, but this increased progressively with treatment. Normalisation using *atp6* or *ubq* resulted in clear induction curves yet exhibited high variability, especially at early time points like 1 h, where we noted greater dispersion. Noteworthy, most normalisation strategies indicated a significant drop in expression at 30 min, disrupting the otherwise upward trend. The induction profile was clearer with *psbB* and *rps1*, though some variability among replicates persisted. Combinations of reference genes produced more reliable results. For instance, using *ubq* + *psbB*, *rps1* + *psbB*, and *ubq* + *rps1* demonstrated well-defined induction curves with clear temporal progression and minimal dispersion among replicates, which enhanced the resolution of expression changes. Additionally, these combinations exhibited reduced statistical variability, consistently showing significant differences across the evaluated time points.

**Figure 6:**
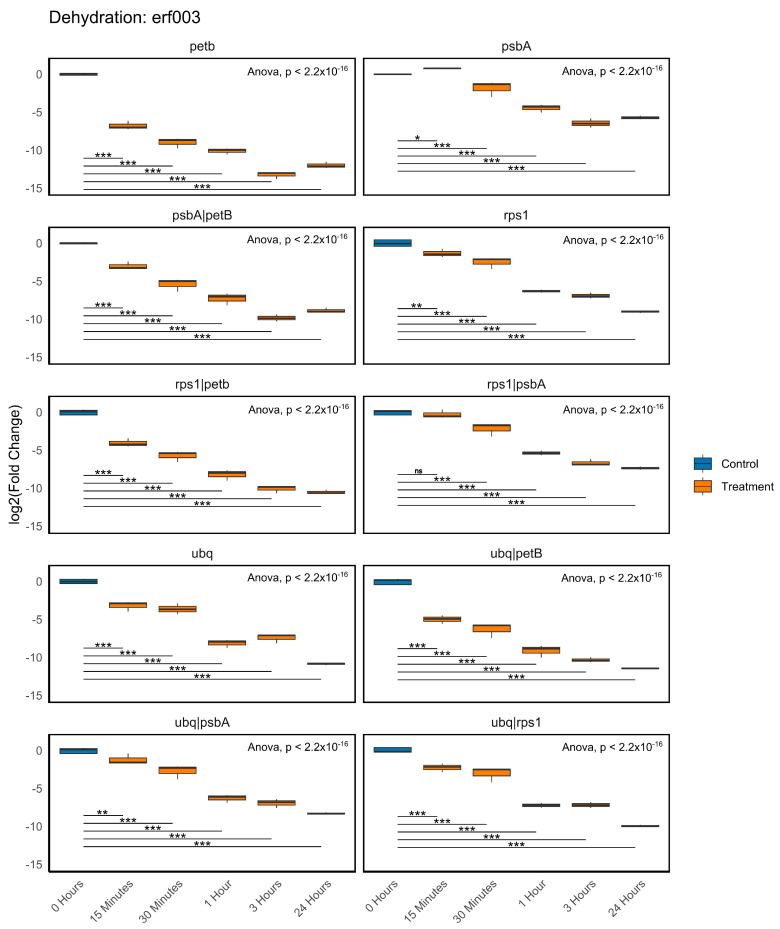
Kinetics of gene expression changes for *erf003* under dehydration stress. Box plots represent the log_2_ (fold change) values obtained using the ΔΔCt method [62] across five time points: 0 h (control), 15 min, 30 min, 1 h, 3 h, and 24 h of dehydration. Each plot corresponds to a different normalisation strategy employing one or two reference genes (*petB*, *psbA*, *rps1*, *ubq*). Technical triplicates were included for each sample. Statistical significance was assessed using one-way ANOVA followed by Tukey’s HSD test. Asterisks denote significance levels: *p* < 0.05 (*), < 0.01 (**), < 0.001 (***); “ns” indicates no significance.

In addition, we analysed the expression profile of *erf003*, a transcription factor found to be repressed under dehydration conditions in our transcriptomes. Figure 6 illustrates the kinetics of *erf003* expression across six time points (0 h, 15 min, 30 min, 1 h, 3 h, and 24 h), employing various normalisation strategies based on individual or combined reference genes (*petB*, *psbA*, *rps1*, *ubq*). Overall, *erf003* displayed a gradual decline in relative expression levels beginning at 15 min of dehydration, with minimum values reached between 3 h and 24 h. This repression trend persisted across most normalisation strategies, though variations were observed in the curve shape and data dispersion depending on the reference gene utilised. For instance, with *petB*, *erf003* repression was evident from 15 min, showing a clear progression and low variability. Normalisation with *psbA* indicated a similar pattern but exhibited greater dispersion at the earlier time points, particularly at 15 min. Using *rps1* for normalisation enabled the detection of gene repression, though the statistical distinction between 0 h and 15 min was somewhat ambiguous. The *ubq* method presented a clear curve but exhibited greater variability at earlier time points. In contrast, combining various reference genes generally provided stronger normalisation. Specifically, combinations like *psbA* + *petB*, *rps1* + *petB*, *ubq* + *petB*, and *ubq* + *psbA* demonstrated a clear and consistent repression of *erf003*, characterised by low dispersion and significant statistical differences across most time points. The combination of *rps1* + *psbA* revealed diminished resolution between 0 h and 15 min (not significant), suggesting a reduced sensitivity for early change detection. Meanwhile, *ubq* + *rps1* delivered progressive and distinctly resolved repression at all measured time points.

Overall, these findings suggest that although single genes can detect the general trend of *hat5* induction or *erf003* repression, combining reference genes greatly enhances normalisation accuracy. This improvement is achieved by minimising experimental noise and ensuring consistency in expression profiles.

## 3. Discussion

RT-qPCR is now considered the gold standard for analysing gene expression because of its high sensitivity, specificity, reproducibility, and speed [40]. Nonetheless, the dependable interpretation of data largely depends on choosing suitable reference genes. Traditionally, housekeeping gene products like ACT, GAPDH, UBQ, eEF-1α, and 18S rRNA have been widely utilised. However, recent research indicates that the expression of these genes can vary significantly depending on factors such as tissue type, treatment, or biological stage, thereby compromising the accuracy of results if not validated for each specific condition or organism [45,56,57,58]. This variability highlights the necessity of validating reference genes for each organism, tissue type, and experimental context. To tackle this issue, we employed a transcriptome-guided approach to identify and validate reference genes for *P. replicatum*, a fully desiccation-tolerant moss, under different abiotic stress conditions.

In this study, 13 reference gene candidates with complete reading frames were selected from transcriptomic datasets due to their stable expression across six conditions: control, dehydration (63% RH), rehydration, 200 mM NaCl, 400 mM sorbitol, and 10 µM ABA. This approach, guided by transcriptomic data, identifies genes with minimal expression variability across different treatments, thereby enhancing the selection of reliable reference genes for RT-qPCR analysis. Similar methodologies have also proven effective in other plant systems, including bryophytes like *P. patens* [58], *S. caninervis* [50], and *S. uncinata* [45], as well as vascular plants such as *Schima superba* [63], *S. lycopersicum* [57], *Allium sativum* L. [64], and *Sorghum sudanense* [65], among others. These studies reinforce the practice of utilising transcriptomic data to preselect candidate genes with stable expression prior to experimental confirmation, particularly in non-model organisms or under specific environmental conditions.

Following this approach, our analysis with BestKeeper and geNorm confirmed that all chosen genes displayed consistent expression across all treatments, supporting their role as internal controls in gene expression research for *P. replicatum* under abiotic stress (Figure 1). Our initial analysis revealed a range of expression levels among the candidates, allowing us to classify them into low and medium transcript abundance [61]. The genes *ubq*, *psbB*, *petB*, *rps1*, and *atp6* showed stable expression across most conditions. In contrast, genes such as *rbcS* (commonly utilised in other systems) and *rh3* showed considerable variability, highlighting the reference gene performance that varies by context. To comprehensively assess gene expression stability, we utilised the RefFinder platform, which integrates four popular algorithms: geNorm, NormFinder, BestKeeper, and the ΔΔCt method. geNorm, an Excel-based tool, determines the average pairwise variation (M value) of a reference gene in comparison to all other genes. A lower M value indicates greater stability in gene expression [49]. Additionally, geNorm determines the ideal number of reference genes needed for accurate normalisation. NormFinder evaluates gene stability by combining intra- and inter-group variations among potential reference genes [38]. BestKeeper is another Excel-based tool that assesses gene expression stability by analysing the variability of Ct values along with the Pearson correlation coefficient (r). This coefficient reflects the relationship between each reference gene and the BestKeeper index, which is derived from the geometric mean of the Ct values of other candidate genes. Genes with a standard deviation (SD) of greater than 1.0 are considered unstable [51]. Finally, RefFinder provides a comprehensive, user-friendly platform that integrates the algorithms geNorm, NormFinder, and BestKeeper, as well as the comparative ΔΔCt method, into one interface. This integration enables researchers to systematically compare and rank potential reference genes by assigning weights based on stability scores from various algorithms. The final ranking is derived from the geometric mean of these weights [52]. The ΔΔCt method is frequently employed in RT-qPCR analysis, allowing for precise comparisons of relative gene expression across different experimental conditions by normalising against reference genes, thus accounting for technical and biological variability [62]. As emphasised in earlier studies [64,66,67,68], employing multiple algorithms improves the reliability of reference gene selection, as they provide complementary statistical insights.

Our combined analysis identified *psbB*, *ubq*, *rps1*, and *petB* as some of the most stable genes, whereas certain genes, like *rh3*, exhibited high stability solely under specific treatments, such as dehydration and sorbitol. These results highlight the necessity of validating reference genes for each particular condition of interest. Identifying *ubq* as a reliable reference gene is consistent with previous research in both bryophytes and vascular plants. For example, UBQ has been identified as a stable gene in *P. patens* during hormonal treatments [58], as well as in *S. lycopersicum* [57] and *Aegilops tauschii* [69] under abiotic stress. Conversely, *rbcS* showed considerable variability, particularly under stress, making it an unsuitable candidate, despite its use in other plant models [70]. This perspective is consistent with observations from Huang et al. [71], who also found *rbcS* inappropriate for normalisation under varying conditions. We propose the innovative use of *psbA*, *psbB*, *petB*, and *atp6* as reference genes; these genes are crucial to the chloroplast genome and are not typically used in RT-qPCR normalisation. Nonetheless, the consistent expression of genes across treatments in *P. replicatum* suggests that chloroplast-encoded genes may be an important alternative in desiccation-tolerant mosses. For instance, Li et al. (2015) identified CDPK and α-TUB2 as the most stable genes under abiotic stress [59], but they did not explore chloroplast genes, highlighting an area that our research begins to investigate. Additionally, several researchers have introduced new non-traditional reference genes. Park et al. (2018) identified the POB1, AKB, and UFD2 genes, which had not been used as references in the Antarctic moss *S. uncinata*, based on transcriptomic analysis [45]. These newly discovered genes demonstrated increased stability under cold and drought stress, emphasising the need to select genes specifically suited to particular organisms and environmental conditions. In *Isatis indigotica*, genes PP2A-4 and TUB4 showed significant stability across different tissues and abiotic stress conditions, whereas traditional reference genes like ACT and GAPDH exhibited notable variability [72]. Our findings, consistent with recent studies, reinforce the idea that no reference gene is universally stable. They also emphasise the need for transcriptome-based screening and empirical validation to identify conserved and novel genes, with contextual stability. It is interesting that genes encoded in chloroplasts (*psbB* and *petB*), mitochondria (*atp6)*, and nuclei (*rps1*, *ubi*) showed relevant expression stability under abiotic stress conditions in the desiccation-tolerant moss *P. replicatum*, especially since the reference genes proposed for other moss species are all nuclear encoded.

Furthermore, the pairwise variation analysis (Vn/Vn+1) conducted with geNorm indicated that only two genes were necessary for reliable normalisation under all tested conditions, as the V2/3 values fell below the 0.15 threshold proposed by Vandesompele et al. (2002) [49]. This discovery reduces the number of required genes and simplifies the technical implementation of RT-qPCR while preserving accuracy. Similar results have been observed in bryophytes, which also recommend two reference genes based on corresponding evaluations [45,58], as well as in the microalga *Chlorella* sp. [68] and various higher plant species [36,41,65,73].

To functionally validate the chosen reference genes, we analysed the expression kinetics of two transcription factors, *hat5* and *erf003*. RT-qPCR experiments were performed at six time points (0 h, 15 min, 30 min, 1 h, 3 h, and 24 h) under dehydration conditions, normalising the data with both individual and combined reference genes. The expression patterns of both transcription factors mirrored the trends seen in our transcriptomic data: *hat5* displayed a progressive and sustained induction, while *erf003* revealed a continuous decline in expression.

Interestingly, a biphasic response in *hat5* expression was noted throughout the dehydration kinetics. Initially, there was a quick rise in *hat5* transcript levels, followed by a decrease at 30 min, and later, a marked increase in mRNA accumulation during extended dehydration. This biphasic pattern was observed consistently across all normalisation strategies. This suggests that the presence of a regulatory checkpoint or a temporary repression event may be biologically significant, which could easily be missed without accurate normalisation. Biphasic gene expression responds to dehydration stress. For example, in barley (*Hordeum vulgare*), certain drought-responsive genes demonstrate a rapid rise initially, then decrease during the early stages of dehydration, pointing to intricate temporal regulation mechanisms [74]. Likewise, in *A. thaliana*, the expression of transcription factors NGATHA1 and NGATHA2 is temporarily inhibited during the early phase of dehydration stress before increasing again, underscoring the dynamic aspects of gene regulation in such environments [75]. The *CpMYB10* transcription factor gene from the desiccation-tolerant plant *Craterostigma plantagineum* also displays a biphasic response to drought, with its overexpression in Arabidopsis leading to enhanced drought and salinity tolerance [76]. Members of the HD-Zip family are recognised for their responses to various abiotic stresses; *hat5*, in particular, has been linked to reactions to salt, cold, toxic metals, and light stimuli. To the best of our knowledge, this is the first documentation of *hat5* being transcriptionally activated by dehydration in a bryophyte.

In contrast, *erf003*, which is part of the ethylene response factor (ERF) subfamily in the AP2/ERF superfamily, showed a consistent decline in expression during dehydration, as confirmed by RT-qPCR. Interestingly, this finding was better illustrated through gene combinations that improved both the sensitivity and statistical significance of the observed alterations. The downregulation of ERF003-like genes in response to abiotic stress has been reported across various angiosperms. In rice, OsERF3 is identified as a negative regulator during drought and salt stress; mutations in its EAR motif have been indicated to modify ethylene biosynthesis and increase tolerance to water scarcity [77]. In tomatoes, it was observed that Sl-ERF.B3 is suppressed under salt and cold stress, and overexpressing it influenced the expression of stress-responsive genes [78]. While specific studies on ERF003 in mosses are lacking, transcriptomic data imply that similar ERF-like factors could play a role in early dehydration responses by modulating hormonal and oxidative stress signalling.

Our findings support the idea that using multiple reference genes reduces individual variations [49]. Recent studies in other species reinforce this approach. For example, in *Salsola ferganica*, the combination of HSC70 and TUB was found to be optimal under water stress induced by PEG [18]. Our results highlight the importance of validating reference genes based on the biological context and experimental conditions. Employing several validated reference genes enhances the reliability of gene expression data and helps detect subtle yet significant expression changes, like the temporary drop in transcript levels seen in *hat5*. While individual genes such as *atp6* or *ubq* revealed general trends, their expression profiles were more inconsistent, especially early on. We propose the combinations of *ubq* + *psbB*, *rps1* + *psbB*, and *ubq* + *rps1* as promising candidates for gene expression studies related to short-term dehydration, improving precision and consistency in measuring differentially regulated genes. This approach is particularly crucial in stress response research, where gene expression dynamics can vary greatly and are time-dependent.

This study on *P. replicatum* represents the first to confirm reference genes in the protonema cells of a fully desiccation-tolerant moss species. It presents a compiled list of reference genes designed for future research on gene expression, especially regarding abiotic stress conditions in protonemal tissue grown in sterile in vitro settings. The identification of novel reference genes in mosses (*psbB*, *rps1*, *atp6*, and *petB*) from transcriptomic data, as demonstrated in this study, offers a reliable and repeatable approach for examining other non-model species with minimal genomic data. Additionally, it emphasises the need to move beyond traditional genes and develop customised strategies to enhance the validity of gene expression research.

## 4. Materials and Methods

### 4.1. Biological Material and Stress Treatments

A monosporic line of *P. replicatum* was used in this work, which was generated from sporophytes collected in Ixtacuixtla, Tlaxcala (19°20´03.3” N, 98°21´59.9” W; altitude: 2159 m) [21]. Protonema tissues were homogenised using an IKA ULTRA TURRAX T10 model dispersing tool (Staufen, Germany) with plastic disposable tips and subcultured every 7 days in PpNH_4_ solid medium under standard growth conditions in a growth room at 24 °C with a 16/8 h light/dark photoperiod and a light intensity of 55 µmol photons m^−2^ s^−1^. For treatments, 7-day-old protonemata were transferred to fresh PpNH_4_ solid medium supplemented with 10 μM ABA, 200 mM NaCl, or 400 mM sorbitol and exposed to these conditions for 3 h. To induce dehydration, the 7-day-old protonema were placed in a desiccation chamber with 63% relative humidity (RH) for 3 h. For rehydration, dehydrated protonema were reintroduced to fresh PpNH_4_ solid medium for 3 h. All treatments, including the control, were conducted simultaneously using the same batch of protonemata tissue. Each condition included three biological replicates, with each replicate consisting of samples that were independently propagated and treated. The treated tissues were immediately frozen after treatment and stored at −80 °C until RNA extraction.

### 4.2. RNA-Seq Analyses and De Novo Transcriptome Assembly

Total RNA was quantified using a Qubit 3.0 Fluorometer (Kingstone, ON, Canada), and RNA integrity was assessed using an Agilent 2100 Bioanalyzer (Santa Clara, CA, USA) (RIN > 7 for all samples). RNA-Seq libraries were performed in triplicate for each condition using the Illumina Stranded Total RNA Prep with Ribo-Zero Plus kit (San Diego, CA, USA). Normalised RNA (paired-end, 2 × 75 bp insert size) was sequenced on the Illumina NovaSeq 6000 SP (San Diego, CA, USA) (300 cycles) platform across five treatments and control, summarising 18 samples. Quality control of the raw sequencing data was assessed at multiple levels. First, overall run quality metrics provided by the sequencer were considered, including a Q30 score above 90% and a passing filter (%PF) rate exceeding 72%, which are standard quality benchmarks for RNA sequencing. Quality control of all raw sequencing data was performed using FastQC program v0.11.8 [79] to assess read quality (Phred scores), GC content, sequence duplication levels, and potential adapter contamination. Low-quality bases (Q < 20) and adapter sequences were trimmed using Trim Galore! v0.6.6. software (https://github.com/FelixKrueger/TrimGalore) (accessed on 20 November 2023) [80] with default parameters, and only reads longer than 50 bp after trimming were retained. Post-trimming quality was re-evaluated with FastQC to confirm improvements in data quality. A de novo transcriptome assembly was generated using Trinity v2.13 [81], with default parameters and a minimum contig length of 200 bp. Transcript abundance was quantified using Kallisto v0.46.1 [82] in pseudoalignment mode. Functional annotation was performed using Trinotate v3.2 [83] and eggNOG-mapper v2.1.5 [84], and open reading frames (ORFs) were predicted using TransDecoder v5.5.0.

### 4.3. Analysis of Differentially Expressed Genes (DEGs)

Statistically, DEGs were identified using three different tools: edgeR [85], limma [86], and DESeq2 [87]. All software returned very similar results in terms of trends, magnitude, and statistical support. Therefore, the mean values of both log2 (fold change) and *p* < 0.05 were used as the criteria for differentially expressed genes for further analysis.

### 4.4. Selection of Candidate Reference Genes

Candidate reference genes were selected based on observed stability across all previously described transcriptomes, evaluated using two established algorithms: BestKeeper [51] and geNorm [49]. This analysis utilised the R package “ctrlGene” (https://cran.r-project.org/web/packages/ctrlGene/) (accessed on 5 November 2024) to integrate these algorithms. The analysis of DEGS resulted in 256 non-differentially expressed genes, which were then used to identify the top candidates through BestKeeper and geNorm, based on parameters including FPM value indices, mean expression value (MV), standard deviation (SD), and coefficient of variation (CV). Additionally, an in silico analysis was performed to obtain the complete open reading frames (ORFs) of the top-ranked reference gene candidates by cross-referencing the Trinity identifiers (IDs) with the entire *P. replicatum* transcriptome sequences. The consensus sequence was generated by aligning each gene isoform sequence with MAFFT, which is integrated in Jalview (https://www.jalview.org) (accessed on 10 December 2024). Only the complete ORF sequences obtained from the ORFfinder web service (https://www.ncbi.nlm.nih.gov/orffinder/) (accessed on 15 January 2025) were utilised for subsequent analysis. The conserved domains served as a second acceptance criterion, using the CD-Search tool (https://www.ncbi.nlm.nih.gov/Structure/cdd/wrpsb.cgi) (accessed on 15 January 2025) from the NCBI Conserved Domain Database.

### 4.5. Primer Design and Evaluation of Candidate Reference Genes

All isoforms of the selected genes were obtained, and multiple sequence alignments were performed using Jalview v2.11.3.0 and SnapGene v6.2.1 to identify the complete ORF sequence. The alignments were then utilised to design the primers using the Oligo Architect (http://www.oligoarchitect.com/LoginServlet) (accessed on 25 January 2025) web service. The criteria for oligo design were as follows: alignment temperature (Ta) of 60–62 °C, oligo length of 18–22 base pairs (bp), GC content between 30–60%, and amplicon length of 100–200 bp. Each primer returned by the program was tested for homology with other genes using BLASTN. To confirm the specificity of the primer, all pairs of primers were initially tested by endpoint PCR using cDNAs from *P. replicatum* as the template, and the amplification product for each gene was verified by 2% agarose gel electrophoresis. The specificity of the primer was confirmed by analysing the melting curve of the RT-qPCR. Three technical replicates of non-template control (NTC) reactions were included in each PCR run for each set of primers.

### 4.6. Total RNA Extraction and cDNA Synthesis

Total RNA was isolated from the specified conditions using a modified TRIzol method. To eliminate traces of genomic DNA, RNase-free DNase I from Qiagen (Hilden, Germany) was employed. RNA concentration and purity were assessed with a DeNovix DS-11FX+ spectrophotometer/fluorometer and analysed by gel electrophoresis. Each RNA sample (5 μg) was reverse transcribed using the SCRIPT cDNA Synthesis Kit (Jena Bioscience) according to the manufacturer’s instructions. Reverse transcription was conducted at 50 °C for 60 min in a final volume of 20 μL, and the enzyme was inactivated at 70 °C for 10 min. Finally, cDNA was stored at −20 °C.

### 4.7. RT-qPCR Analysis

RT-qPCR was performed using the qPCRSybrMaster kit (Jena Bioscience; Jena, Germany), specifically the Jenna mix kit, in 10 μL reactions following the supplier’s instructions. This included 0.5 μL of a dilution (1:10, 1:5, and 1:2) of cDNA, 0.1 μM of each oligonucleotide, 5 μL of SybrMaster, and 3.7 µL of PCR-grade water. The amplified signals were continuously monitored with the Bio-Rad CFX96 real-time thermocycler. The amplification protocol consisted of 5 min of denaturation and enzyme activation at 95 °C, followed by 34 cycles at 95 °C for 15 s, 60 °C for 30 s, and 72 °C for 15 s. Three biological replicates were employed for each sample, with three technical replicates.

### 4.8. RT-qPCR Data Analysis of Reference Gene Stability

Quantification cycle (Cq) values indicating gene expression levels were determined by RT-qPCR amplification for each candidate reference gene from all RNA samples. The RT-qPCR Cq value of each gene was obtained by calculating the arithmetic mean from four technical and three biological replicates. To rank the reference genes based on their expression stability under different experimental conditions, we employed four distinct algorithms: GeNorm, BestKeeper, NormFinder, and ΔΔCt. The Cq value of each reference gene was converted into an input file according to the respective software manuals. For geNorm and NormFinder analysis, the raw Cq values were converted into relative value using the formula Q = 2 − ΔCq, in which ΔCq equals each corresponding Cq minus the minimum Cq. These values were then submitted to the Excel-based geNorm applet to calculate the expression stability value (M), which is described as the average pairwise variation (V) of a candidate reference gene. Also, NormFinder was used to calculate the stability value using an ANOVA-based model to consider intra-group and inter-group variation in the candidate reference genes, with the lowest value representing the highest stability. For BestKeeper, the raw data (Cq values) were used to calculate the coefficients of variance (CV) and the standard deviation (SD), with the lowest CV representing the highest stability. To calculate the ΔΔCt, the Livak method was employed. Finally, the stability rankings of 13 candidate reference genes were combined to create a comprehensive ranking. This was achieved by calculating the geometric mean derived from the results obtained using the four methods.

### 4.9. Normalisation of the Expression of P. replicatum Dehydration-Responsive Genes in RT-qPCR Experiments

To normalise the expression of differentially expressed genes in response to abiotic stress, the reference genes identified in this study were employed in RT-qPCR experiments. For this purpose, we selected two transcription factors that are differentially expressed during dehydration. According to our transcriptomic data, the transcript levels for the Homeobox-leucine zipper protein-like (*hat5*) protein increased in response to dehydration, whereas the levels of ethylene-responsive transcription factor 03 (*erf003*) decreased. Both transcription factors were chosen as target genes to normalise their expression using pairwise combinations of the four top-ranked reference genes under dehydration conditions at various time points.

## 5. Conclusions

This study marks the first identification and validation of reference genes appropriate for gene expression analysis by RT-qPCR of in vitro cultured protonemata of any fully desiccation-tolerant moss species, specifically *P. replicatum*. By analysing transcriptomic data alongside four well-established algorithms (geNorm, NormFinder, BestKeeper, and RefFinder), we identified the genes *ubq*, *psbB*, *rps1*, *atp6*, and *petB* as the most stable across six experimental conditions. A pairwise variation analysis determined that utilising just two reference genes was adequate for reliable normalisation. Classic reference genes such as *ubi* and *rbcS* were also found. Remarkably, we discovered reference genes not previously associated with mosses, specifically *psbB*, *rps1*, *atp6*, and *petB*. Notably, genes encoded in chloroplasts (*psbB* and *petB*), mitochondria (*atp6*), and nuclei (*rps1*, *ubi*) demonstrated significant expression stability under abiotic stress in the desiccation-tolerant moss *P. replicatum*, which was remarkable since the reference genes typically proposed for other moss species are solely nuclear-encoded. These discoveries offer essential tools for gene expression research in *P. replicatum* and provide a replicable approach for other non-model species. The functional validation conducted with target genes emphasises the importance of empirically assessing each gene combination and confirms that stability should be contextualised within each biological system. In conclusion, this research profoundly enhances the utility of *P. replicatum* as an emerging model for investigating abiotic stress responses in bryophytes. It marks the first documentation of validated reference genes for RT-qPCR gene expression research in the protonemal tissue of a fully desiccation-tolerant moss under abiotic stress conditions.

## Figures and Tables

**Table 1:** Primers designed for RT-qPCR experiments.

Category	ID	Gene	Primers Forward 5′ a 3′	Primers Reverse 5′ a 3′	Amplicon (bp)
Putative reference genes	TRINITY_DN1938_c133_g1	*psbA*	CATCGTAGCTGCTCATGGTTAC	GAAAGCCATTGTGCTGATACCT	145
	TRINITY_DN9694_c9_g1	*psbB*	TGATCCTTTTGTTCCAGGAGGA	CAGTTACACCATTGCCCATACG	178
	TRINITY_DN1103_c0_g1	*atp6*	CCCTTGTCGTTAGCACCCTTC	GTCCAAGCAAACCCGCTTAGAA	140
	TRINITY_DN4025_c0_g2_i1	*clpP*	TCTCCTGGTGGAGCTGTATTAG	AAAGCAAAGCCGCAAAGC	140
	TRINITY_DN798_c0_g1_i4	*rps1*	TGGCAATATCGTGGTAAAAGAGA	GCAGGTGTTTCCGCTTTG	174
	TRINITY_DN269_c0_g1_i28	*rbcS*	ATGATTCAGCAGAACCTG	ATCCACATGGTCCAGTAG	104
	TRINITY_DN1154_c1_g1	*ubq*	TCAAGCACAAGAAGAAGAAGG	ATCAGGGTTTGGGCACTC	116
	TRINITY_DN2574_c0_g1	*vcs*	CTGTTGAGACTTGTGAAGAC	GCGGAGCAATCTGTTAAG	148
	TRINITY_DN224_c0_g1	*scyl2a*	CCATCATACTCGCGGACCATC	TCGCCGCTGTTAGAGTA	174
	TRINITY_DN5925_c0_g1	*gpc1*	CGGCGGAGATTCAGTAGA	CATGGAGTTGCTCATCAGATT	137
	TRINITY_DN5657_c0_g1	*rh3*	CAATGTCGGCAAGATTCGTA	CACCAACTTCGGCAACTT	148
	TRINITY_DN717_c2_g1_i1	*petB*	AGGAGGCATCACCTTAACTTGTTT	CCATCATACTCGCGGACCATC	173
	TRINITY_DN5651_c0_g3	*psbE*	AGGAGAGCGCCCTTTTGCTGATA	AGGCACTTCTTACCGGCTTACTGT	178
Abiotic stress responsive genes	TRINITY_DN80_c0_g3	*hat5*	TGGAAGACGAAGCAACTG	AGTGGACTCACCTGACAG	138
	TRINITY_DN2254_c0_g1	*erf003*	CTTCTTCGACTGCCAGAT	GAAGGAGTCTTGCGTGAA	100

## Data Availability

The sequence of the genes used in the RT-qPCR experiments will be available upon request.

## Supplementary Materials

The following supporting information can be downloaded at: https://www.mdpi.com/article/10.3390/plants14121752/s1, File S1: Full transcript sequences of selected genes.
